# Assessment of patient-reported adverse events after discharge from hospital in RCTs in gastrointestinal cancer surgery: is there sufficient coverage in existing EORTC questionnaires?

**DOI:** 10.1186/1745-6215-16-S2-P48

**Published:** 2015-11-16

**Authors:** Kerry Avery, Elaine O’Connell Francischetto, Galina Velikova, Jane Blazeby

**Affiliations:** 1 School of Social and Community Medicine, University of Bristol, Bristol, UK; 2 Leeds Institute of Cancer Studies and Pathology, University of Leeds, Leeds, UK

## Background

There is a need for assessment of adverse event (AEs) after hospital discharge in RCTs in surgery. Currently, measures to assess patient-reported AEs are limited. The EORTC quality of life questionnaires (QLQ) cover AEs and functions.

This research aimed to assess if AEs covered in EORTC QLQ upper gastrointestinal (GI) modules could be used to assess AEs post-discharge after surgery.

## Methods

Questionnaire items and scales in the EORTC QLQ-C30 and the five validated upper GI modules were analysed. Items not relevant to surgical AEs were excluded. Items and scales with overlapping content were scrutinised. Published validation papers were reviewed for associations between module and core questionnaire scales and items. Semi-structured interviews were then conducted with patients to ensure the questionnaire covered relevant AEs.

## Results

There were 134 items (72 scales/single items) identified from the C30 and five modules. Methods initially excluded 17 items/scales relevant only to chemoradiation treatment or psycho-social issues. There were 13 overlapping scales/items in the five modules, so one scale/item was selected. The disease-specific pain and fatigue scales were moderately correlated with scales in the C30, so only the C30 scales were retained. The shorter physical functioning scale in the EORTC QLQ-C15-PAL was used. The final questionnaire comprised of 34 items. Interviews with 18 patients showed sufficient coverage except for wound-related problems.

## Conclusion

Existing validated disease-specific EORTC modules can be utilised to form an upper GI questionnaire to measure AEs in surgical patients in RCTs. Further work is under way to identify clinically important thresholds.

“This abstract presents independent research funded by the National Institute for Health Research (NIHR) under its Programme Grants for Applied Research Programme (Reference Number RP-PG-0611-20008). The views expressed are those of the author(s) and not necessarily those of the NHS, the NIHR or the Department of Health. We also acknowledge support from the MRC ConDuCT-II Hub.”

